# A shift in the virulence potential of *Corynebacterium pseudotuberculosis* biovar *ovis* after passage in a murine host demonstrated through comparative proteomics

**DOI:** 10.1186/s12866-017-0925-6

**Published:** 2017-03-22

**Authors:** Wanderson M. Silva, Fernanda A. Dorella, Siomar C. Soares, Gustavo H. M. F. Souza, Thiago L. P. Castro, Núbia Seyffert, Henrique Figueiredo, Anderson Miyoshi, Yves Le Loir, Artur Silva, Vasco Azevedo

**Affiliations:** 1 Departamento de Biologia Geral, Instituto de Ciências Biológicas, Universidade Federal de Minas Gerais, Belo Horizonte, Minas Gerais Brazil; 2 Instituto de Ciências Biológicas, Universidade Federal do Pará, Guamá, Belém, Pará Brazil; 3 Waters Corporation, Waters Technologies Brazil, MS Applications Laboratory, Alphaville, São Paulo, Brazil; 4 INRA, UMR1253 STLO, 35042 Rennes, France; 5 Agrocampus Ouest, UMR1253 STLO, 35042 Rennes, France; 6 Aquacen, Escola de Veterinária, Universidade Federal de Minas Gerais, Belo Horizonte, Brazil

**Keywords:** *Corynebacterium pseudotuberculosis*, Bacterial label-free proteomic, Caseous lymphadenitis, Bacterial virulence, Serial passage, Extracellular proteins

## Abstract

**Background:**

*Corynebacterium pseudotuberculosis* biovar *ovis,* a facultative intracellular pathogen, is the etiologic agent of caseous lymphadenitis in small ruminants. During the infection process, *C. pseudotuberculosis* changes its gene expression to resist different types of stresses and to evade the immune system of the host. However, factors contributing to the infectious process of this pathogen are still poorly documented. To better understand the *C. pseudotuberculosis* infection process and to identify potential factors which could be involved in its virulence, experimental infection was carried out in a murine model using the strain 1002_*ovis* and followed by a comparative proteomic analysis of the strain before and after passage.

**Results:**

The experimental infection assays revealed that strain 1002*_ovis* exhibits low virulence potential. However, the strain recovered from the spleen of infected mice and used in a new infection challenge showed a dramatic change in its virulence potential. Label-free proteomic analysis of the culture supernatants of strain 1002_*ovis* before and after passage in mice revealed that 118 proteins were differentially expressed. The proteome exclusive to the recovered strain contained important virulence factors such as CP40 proteinase and phospholipase D exotoxin, the major virulence factor of *C. pseudotuberculosis*. Also, the proteome from recovered condition revealed different classes of proteins involved in detoxification processes, pathogenesis and export pathways, indicating the presence of distinct mechanisms that could contribute in the infectious process of this pathogen.

**Conclusions:**

This study shows that *C. pseudotuberculosis* modifies its proteomic profile in the laboratory versus infection conditions and adapts to the host context during the infection process. The screening proteomic performed us enable identify known virulence factors, as well as potential proteins that could be related to virulence this pathogen. These results enhance our understanding of the factors that might influence in the virulence of *C. pseudotuberculosis*.

**Electronic supplementary material:**

The online version of this article (doi:10.1186/s12866-017-0925-6) contains supplementary material, which is available to authorized users.

## Background

*Corynebacterium pseudotuberculosis* biovar *ovis* is a Gram-positive facultative intracellular pathogen. It is the etiologic agent of Caseous Lymphadenitis (CLA) in small ruminants, a disease characterized by abscess formation in lymph nodes and internal organs [1]. Cases of human infection caused by *C. pseudotuberculosis* have been reported and are associated with occupational exposure [1]. CLA is globally distributed and causes significant economic losses in goats, and sheep herds [2]. The pathogenic process of *C. pseudotuberculosis* in the host comprises two phases: (i) initial colonization and replication in lymph nodes that drain the site of infection, which is associated with pyogranuloma formation, and (ii) a secondary cycle of replication and dissemination via the lymphatic or circulatory systems. This dissemination is promoted by the action of phospholipase D (PLD) exotoxin, the major virulence factor of *C. pseudotuberculosis,* which allows this pathogen to contaminate visceral organs and lymph nodes, where it ultimately induces lesion formation [3–5].

Exported proteins reportedly favor the infection process in pathogenic bacteria; this class of proteins is involved in adhesion and invasion of host cells, nutrient acquisition, toxicity, and in the evasion of the host immune system [6]. Different strategies like the transposon mutagenesis have been adopted to identify *C. pseudotuberculosis* biovar *ovis* exported proteins [7]. Additionally, comparative proteomics has been applied to characterize the extracellular proteome of *C. pseudotuberculosis* biovar *ovis,* as well as, the extracellular immunoproteome (strains C231_*ovis* and 1002_*ovis*) [8–11]. In these studies, some proteins of the strain 1002*_ovis*, suspected to be virulence factors, were not detected suggesting this strain presents a low virulence. The surface proteome of *C. pseudotuberculosis* biovar *ovis* was also characterized using bacterial strains isolated from the lymph nodes of naturally infected sheep. This proteomic analysis allowed the identification of proteins that could favor the survival of this pathogen during the chronic phase of CLA [12].

The experimental passage of bacterial pathogens through in vitro or in an in vivo model is a strategy that has been applied to evaluate the virulence potential of several pathogens. By generating a confrontation between the pathogen and the dynamic network of host factors, including the immune system components, it helps to identify bacterial factors involved in virulence [12–19]. In this study, the strain 1002*_ovis* was experimentally inoculated in mice [20, 21] to identify factors which could contribute to virulence in *C. pseudotuberculosis* biovar *ovis.* Comparative proteomics of the culture supernatant from this strain collected before and after the experimental passage in mice was carried out to identify factors that might contribute to virulence of 1002_*ovis*.

## Methods

### Bacterial strains and growth conditions

The *C. pseudotuberculosis* biovar *ovis* strain 1002 (1002_*ovis*) was isolated from a goat in Brazil; this strain was cultivated under standard conditions in brain–heart infusion broth (BHI-HiMedia Laboratories Pvt. Ltd., India) at 37 °C. When necessary, 1.5% of agar was added to the medium for a solid culture. For extracellular proteomic analyses, 1002*_ovis* was grown in a chemically defined medium (CDM) [(Na_2_HPO_4__7H_2_O (12.93 g/L), KH_2_PO4 (2.55 g/L), NH_4_Cl (1 g/L), MgSO_4__7H_2_O (0.20 g/L), CaCl_2_ (0.02 g/L) and 0.05% (v/v) Tween 80], 4% (v/v) MEM Vitamins Solution (Invitrogen, Gaithersburg, MD, USA), 1% (v/v) MEM Amino Acids Solution (Invitrogen), 1% (v/v) MEM Non-Essential Amino Acids Solution (Invitrogen), and 1.2% (w/v) glucose at 37 °C [22].

### Experimental infection of strain 1002_*ovis* in a murine model (in vivo assay)

The standardization of the parameters for infection was performed according to Moraes et al. [20] and Ribeiro et al. [21]. Female BALB/c mice between six and eight weeks old were used in all experiments. They were provided by the Animal Care Facility of the Biological Sciences Institute from the Federal University of Minas Gerais and were handled by the guidelines of the UFMG Ethics Committee on Animal Testing (Permit Number: CETEA 103/2011). For the bacterial passage assay using the murine model, two groups of three mice each was infected via intraperitoneal injection with 10^6^ colony forming units (CFU) of strain 1002_*ovis*. Thirty-six hours after infection, all animals were sacrificed. Their spleens were aseptically removed to recover the bacterial strain, as described below: the spleen removed from each animal was then, individually macerated in sterile saline solution (0.9% NaCl_2_), seeded onto BHI agar plates and incubated for 48 h at 37 °C. Subsequently, one recovered bacterial colony was cultured in BHI broth. The recovered bacteria were then referred to as Recovered (Rc). For the bacterial virulence assay, we used the freshly recovered bacteria and bacteria that did not contact the murine host as a control, which is referred to as Control (Ct). Groups of five mice were infected with Rc and Ct, via intraperitoneal injection of a suspension containing 10^6^ CFU or 10^5^ CFU. The animals’ survival rates were calculated and represented in GraphPad Prism v.5.0 (GraphPad Software, San Diego, CA, USA) using the Kaplan-Meier survival function. The results of 1002_*ovis* CFU count in the organs were calculated using the two-way ANOVA test.

### Preparation of proteins from culture filtrates for proteome analysis

For proteomic analysis, the Ct and Rc (three independently recovered colonies) that was obtained from infected mice spleens as described above were grown in CDM at OD_600_ = 0.8. The cultures were then centrifuged for 20 min at 2700 × g. The supernatants were then filtered using 0.22-μm filters, 30% (w/v) ammonium sulfate was added to the samples, and the pH of the mixtures was adjusted to 4.0. Next, 20 mL N-butanol was added to each sample. The samples were centrifuged for 10 min at 1350 xg and 4 °C. The interfacial precipitate was collected and resuspended in 1 mL of 20 mM Tris–HCl pH 7.2 [23]. Finally the concentration protein was determined by Bradford method [24].

### 2D-PAGE electrophoresis and Mass Spectrometry

The 2-DE procedure and in-gel protein digestion were performed as described previously [9, 10]. Approximately 300 μg of the protein extract from of each condition was dissolved in rehydration buffer (Urea 7 M, thiourea 2 M, CHAPS 2%, Tris–HCl 40 mM, bromophenol blue 0.002%, DTT 75 mM, IPG Buffer 1%). Samples were applied to 18 cm pH 3–10 N.L strips (GE Healthcare, Pittsburgh, USA). Isoelectric focusing (IEF) was performed using the apparatus IPGphor 2 (GE Healthcare) under the following voltages: 100 V 1 h, 500 V 2 h, 1000 V 2 h, 10,000 V 3 h, 10,000 V 6 h, 500 V 4 h. The IPG strips were placed on 12% acrylamide/bis acrylamide gels in an Ettan DaltSix II system (GE Healthcare). The gels were stained with Coomassie Blue G-250 staining solution, and 2-DE gels were scanned using an Image Scanner (GE Healthcare). The Image Master 2D Platinum 7 (GE Healthcare) software was used to analyze the generated images and all spots were matched and analyzed by gel-to-gel comparison. The quantification of the spots was calculated according percentage volume (% Vol) and spots with reproducible changes in abundance were considered to be differentially expressed. Protein spots were excised from the gels, and in-gel digestion was carried out using trypsin enzyme (Promega, Sequencing Grade Modified Trypsin, Madison, WI, USA). The peptides were then desalted and concentrated using ZIP TIP C18 tips (Eppendorf).

The samples were subsequently analyzed for MS and MS/MS modes, using an MALDI-TOF/TOF mass spectrometer Autoflex IIITM (Bruker Daltonics, Billerica USA). The equipment was controlled in a positive/reflector way using the Flex-ControlTM software (Brucker Daltonics). External calibration was performed using peptide standards samples (angiotensin II, angiotensin I, substance P, bombesin, ACTH clip 1–17, ACTH clip 18–39, somatostatin 28, bradykinin Fragment 1–7, Renin Substrate tetra decapeptide porcine) (Bruker Daltonics). The peptides were added to the alpha-cyano-4-hydroxycinnamic acid matrix, applied on an Anchor-ChipTM 600 plate (Brucker Daltonics) and analyzed by Autoflex III. The search parameters were as follows: enzyme; trypsin; fixed modification, carbamidomethylation (Cys); variable modifications, oxidation (Met); mass values, monoisotopic; maximum missed cleavages, 1; and peptide mass tolerance of 0.005% Da (50 ppm). The results obtained by MS/MS were used to identify proteins utilizing the MASCOT_ (http://www.matrixscience.com) program and compared with the genomic data of the Actinobacteria class deposited in the NCBI nr database.

### 2D nanoUPLC-HDMSE data acquisition and Data Processing

The protein extracts from three biological replicates of each condition were concentrated using spin columns with a 10 kDa threshold (Millipore, Billerica, MA, USA) to perform the label-free proteomic analysis. The protein was denatured (0.1% *Rapi*GEST SF at 60 °C for 15 min) (Waters, Milford, CA, USA), reduced (10 mM DTT), alkylated (10 mM iodoacetamide) and enzymatically digested with trypsin (Promega). The digestion process was stopped by adding 10 μL of 5% TFA (Fluka, Buchs, Germany), and glycogen phosphorylase (Sigma, Aldrich, P00489) was added to the digested samples after digest at 20 fmol.uL^−1^ as an internal standard for normalization. Each replicate was injected using a two-dimensional reversed phase (2D RPxRP) nanoUPLC-MS (Nano Ultra Performance Liquid Chromatography Mass Spectrometry) approach with 171 multiplexed high definition mass spectrometry (HDMSE) label-free quantitation [25]. Qualitative and quantitative experiments were performed using both a 1 h reversed phase gradient from 7% to 40% (v/v) acetonitrile (0.1% v/v formic acid) at 500 nL.min^−1^ and a nanoACQUITY UPLC 2D RPxRP Technology system [26]. A nanoACQUITY 174 UPLC HSS T3 1.8 μm, 75 μm × 15 cm column (pH 3) was used with an RP XBridge BEH130 C18 5 μm 300 μm x 50 mm nanoflow column (pH 10). Typical on-column sample loads were 250 ng of the total protein digests for each of the 5 fractions (250 ng/fraction/load). All analyses were performed using nano electrospray ionization in the positive ion mode nanoESI (+) and a NanoLockSpray (Waters, Manchester, UK) ionization source. The mass spectrometer was calibrated using an MS/MS spectrum of [Glu1]-Fibrinopeptide B human (Glu-Fib) solution (100 fmol.uL-1) delivered through the NanoLockSpray source reference sprayer. Multiplexed data-independent (DIA) scanning with additional specificity and selectivity for non-linear ‘T-wave’ ion mobility (HDMSE) experiments were performed using a Synapt G2-S HDMS mass spectrometer (Waters, Manchester, UK).

Following the identification of proteins, the quantitative data were packaged using dedicated algorithms [27] and searching against a database with default parameters to account for ions [28]. The databases used were reversed on-the-fly during the database queries and appended to the original database to assess the false positive rate during identification. For proper spectra processing and database searching conditions, the ProteinLynxGlobalServer v.2.5.2 (PLGS) with IdentityE and ExpressionE informatics v.2.5.2 (Waters, Manchester, UK) was used. UniProtKB (release 2013_01) with manually reviewed annotations was used, and the search conditions were based on taxonomy (*Corynebacterium pseudotuberculosis*). One missed cleavage by trypsin was allowed be up to 1 and various modifications as carbamidomethyl (C), Acetyl N terminal, phosphoryl (STY) and oxidation (M) were allowed [29]. The proteins collected were organized by the PLGS ExpressionE tool algorithm into a statistically significant list that corresponded to higher or lower regulation ratios between the different groups. For protein quantitation, we used the PLGS v2.5.2 software with the IdentifyE algorithm using the Hi3 methodology. The search threshold to accept each spectrum was the default value for a false discovery rate 4%. The quantitation values were averaged over all samples, and the standard deviations of *p* < 0.05, which were determined using the ExpressionE software, refer to the differences between biological replicates.

### Bioinformatic analysis

The proteins identified in 1002_*ovis* under both conditions were analyzed using the following prediction tools: SecretomeP 2.0 server, to predict proteins exported from non-classical systems (positive prediction score greater than to 0.5) [30] and PIPs software, to predict proteins in the pathogenicity islands [31]. Gene ontology (GO) functional annotations were generated using the Blast2GO tool [32].

## Results

**Fig. 1:**
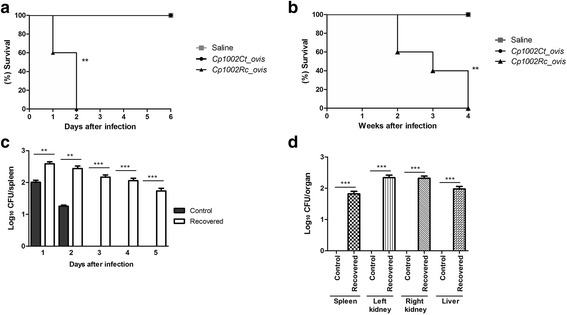
Survival of Balb/C mice infected with strain 1002_*ovis*. **a** The survival rate was measured to determine the virulence profile of strain 1002*_ovis* control and recovered in mice infected with 10^6^ CFU of bacteria Ct = control condition, Rc recovered condition. **b** Survival rates of mice infected with 10^5^ CFU of Ct and Rc. **c** CFU in the spleen of BALB/c mice infected with control and recovered condition for the first five days of infection. **d** CFU in the different organs (spleen, left kidney, right kidney and liver) of BALB/c mice infected with control and recovered condition after four weeks of infection. The mortality rates were measured daily. Results represent three independent experiments. *P* values of <0.05 were considered to be statistically significant, and asterisks indicate statistically significant differences

The main objective of this study was to assay the virulence of 1002_*ovis* in a murine model after passage through mice. We thus carried out an in vivo survival assay using BALB/c mice infected with bacteria that did not contact with murine model (Ct) and bacteria recovered (Rc) from mice spleens. In this assay using an infection inoculum of 10^6^ CFU, all the animals infected with Rc died within 48 h after infection (Fig. 1a). On the other hand, the control group, infected with Ct*,* survived the evaluation period (6 days). Similarly, in an assay with a lower infective dose (10^5^ CFU), a 100% mortality was observed four weeks post infection with the recovered bacteria (Fig. 1b). Comparison of the Ct and Rc numbers isolated from the spleen within five days of infection (Fig. 1c) showed that the serial passage process affected the potential for spleen colonization during the infection. After four weeks of infection in the assay with 10^5^ CFU, bacteria were isolated from the spleen, liver, left and right kidney, only in mice infected with Rc (Fig. 1d). Finally, regarding the clinical signs, in the assay using 10^5^ CFU, caseous lesions were detected in different organs (liver, left kidney and right kidney) of all the animals infected only with Rc (data not shown). Altogether, these results showed that the serial passage process in a murine model increased the virulence potential of strain 1002_*ovis*. In addition, these results confirmed the low virulence of this strain, which was previously suggested based on the composition of its extracellular proteome [8–10].

**Fig. 2:**
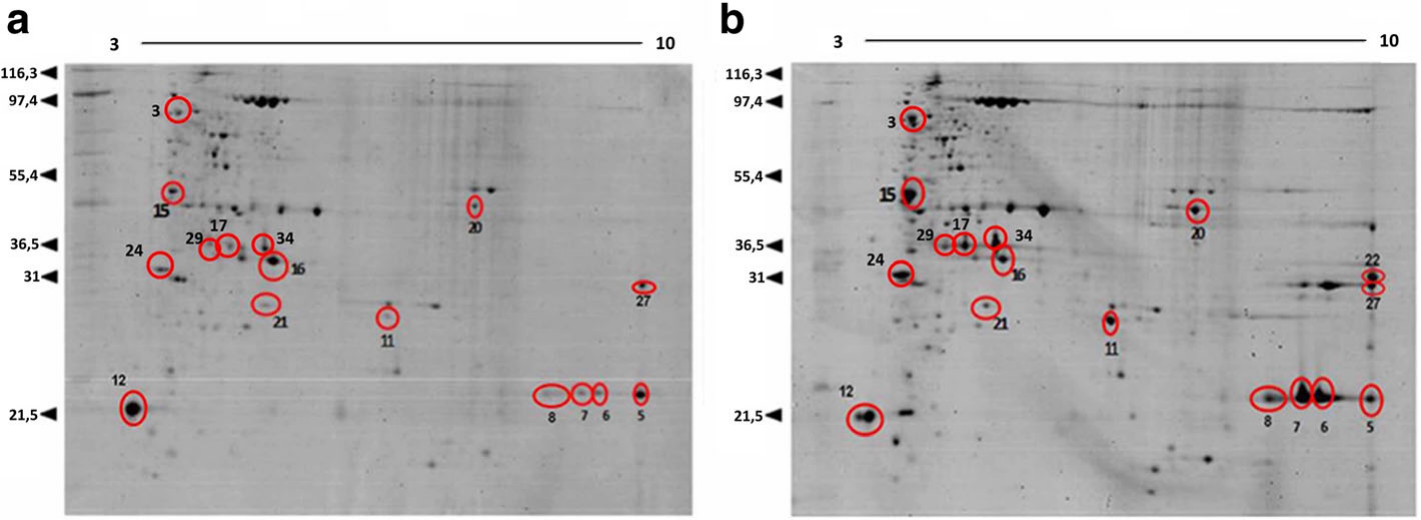
Two-dimensional electrophoresis of the extracellular proteins 1002_*ovis* after following passage process: **a** Control condition. **b** Recovered condition. Red circle: spot proteins identified by MS/MS

**Fig. 3:**
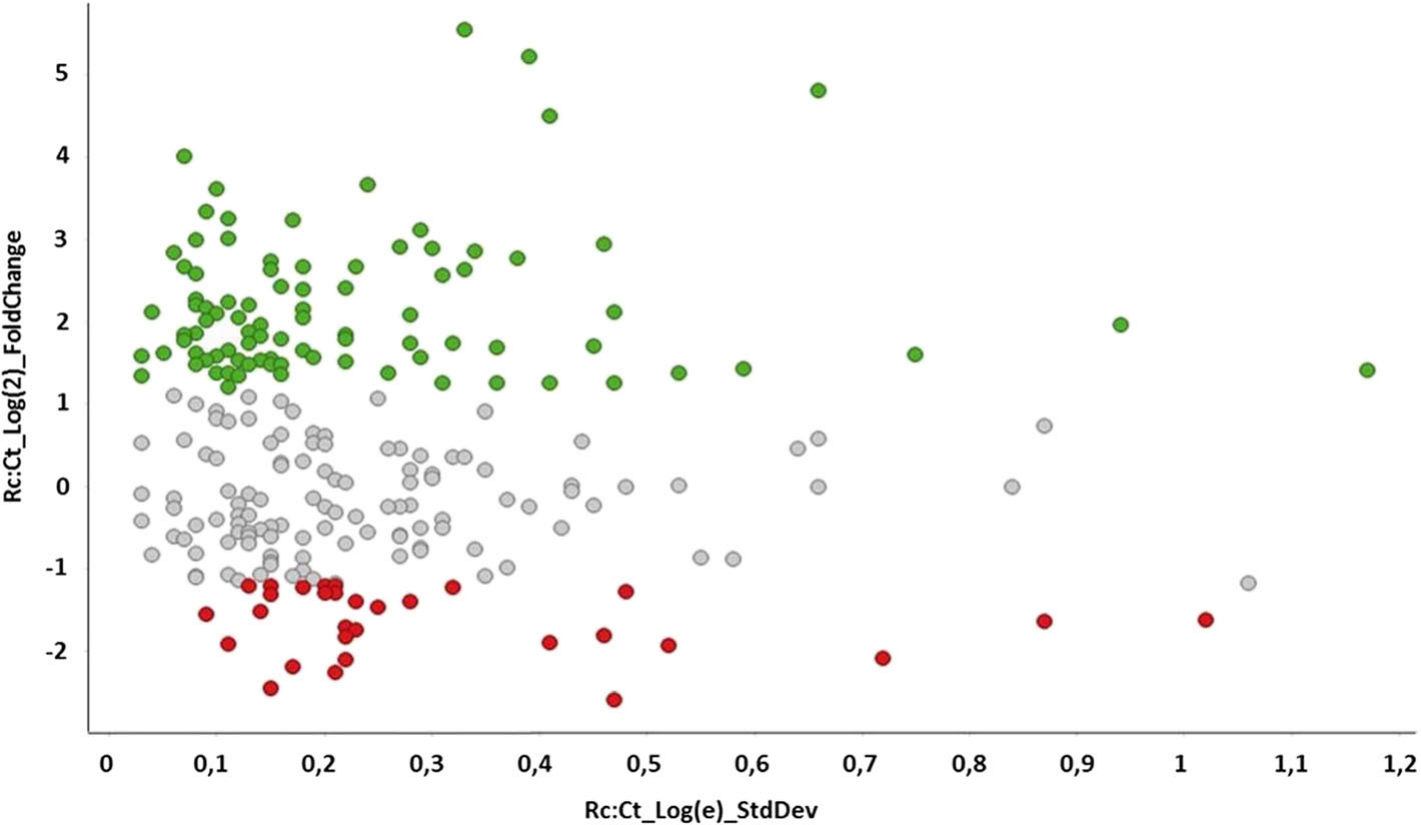
Volcano Plot show Log(2) Fold Change of the differentially expressed proteins detected by label-free proteomics between the recovered and control condition. Green: Up-regulated proteins; Grey: unchanged proteins; Red: Down-regulated proteins

After passage in BALB/c mice, a dramatic change in the virulence potential of strain 1002*_ovis* was observed. We thus hypothesized that this phenotypic change was visible at the proteome level since *C. pseudotuberculosis* virulence relies on the production of a proteinaceous virulence factor. Thus, considering the importance of extracellular proteins for bacterial virulence, the proteomic analysis was conducted on the extracellular proteomes of 1002*_ovis* recovered from infected mice spleens in comparison to the control condition, using two proteomics approaches: 2-DE and 2D nanoUPLC-HDMS^E^. The electrophoretic resolution of the extracellular protein extract of Ct and Rc condition allowed the visualization of spots distributed over pH 3–10 (Fig. 2). A total of 14 spots were found to be differentially expressed between Ct and Rc condition, these spots were excised out of the gel, and identified by MS/MS (Table 1). In the LC/MS analysis, we used the label-free quantitative proteomic to evaluate the relative difference between the proteome of Rc and Ct condition. In this analysis, only proteins which presented *p* < 0.05 and differential expression (log2 ratios) equal or greater than a factor of 1.2 were considered, as described previously [33]. We detected a total of 118 expressed differentially proteins, between Ct and Rc condition (Fig. 3) (Table 2 and Additional file 1). Also, 48 proteins were assigned only to Ct (Additional file 2) and 32 proteins were exclusive to Rc (Table 3) The information about sequence coverage and a number of identified peptides for each protein sequence identified, as well as the information about the native peptide are available at Additional file 3: Table S3.

**Table 1:** List of proteins identified in 1002_*ovis* control and recovered by 2D-PAGE-MS/MS

Spot	Description	Accession	MW(kDa)/p.I	Peptides Number	Mascot Score	Molecular function
5, 6, 7	Hypothetical protein	ADL20032	24.30/9.24	2	189	Unknown function
11,29	Trypsin-like serine protease	ADL20653	25.72/6.49	2	96	Serine-type endopeptidase activity
15	Hypothetical protein	ADL21714	42.04/5.22	4	159	Catalytic activity
20,34	Corynomycolyl transferase	ADL21610	41.80/7.05	2	58	Transferase activity
16	Cytochrome c oxidase sub II	ADL21302	40.33/6.03	2	96	Cytochrome-c oxidase activity
21	Hypothetical protein	ADL21914	12.30/5.04	2	53	Unknown function
12	Hypothetical protein	ADL19922	19.86/4.30	2	145	Calcium ion binding
8	Hypothetical protein	ADL09626	24.30/9.24	3	228	Unknown
27	Hypothetical protein	ADL20508	31.62/9.52	2	66	Unknown
22	Phospholipase D	ADL19935	34.09/8.91	4	286	Sphingomyelin phosphodiesterase D activity
3	Enolase	ADL20605	45.17/4.68	3	271	Phosphopyruvate hydratase activity
17	Trehalose corynomycolyl transferase B	ADL21814	36.67/6.90	5	245	Transferase activity, transferring acyl groups other than
24	Hypothetical protein	ADL21714	40.90/5.05	3	190	Catalytic activity

**Table 2:** Proteins differentially produced among the recovered and control condition

Accession	Description	Score	Fold Change_Log_(2)_^a^	SecretomeP
Transport				
D9Q5H9_CORP1	Periplasmic binding protein LacI	5601,78	3,26	0.612642
D9Q6G4_CORP1	Oligopeptide binding protein oppA^b^	4120,1	3,00	0.892226
D9Q4T5_CORP1	ABC transporter domain containing ATP	1264,05	2,57	0.084974
D9Q7K5_CORP1	Oligopeptide binding protein oppA^b^	33697,17	2,11	0.873687
D9Q5B8_CORP1	Oligopeptide binding protein oppA^b^	852,88	1,88	0.849217
D9Q6C3_CORP1	ABC type metal ion transport system permease	650,43	1,59	0.078043
D9Q796_CORP1	Glutamate binding protein GluB	6254,68	−1,46	0.840325
D9Q7W9_CORP1	Iron(3+)-hydroxamate-binding protein fhuD	2774,62	−1,62	0.824030
Cell division				
D9Q7G1_CORP1	Septum formation initiator protein	2071,46	1,38	0.551153
Cell adhesion				
D9Q5H7_CORP1	Hypothetical protein	115906,3	1,51	0.840443
DNA synthesis and repair				
D9Q7J1_CORP1	GTP binding protein YchF	3487,98	2,68	0.042575
D9Q5F7_CORP1	Chromosome partitioning protein ParB^b^	2467,24	2,44	0.052395
D9Q5G6_CORP1	DNA polymerase III subunit beta	1907,74	1,80	0.071008
D9Q5V6_CORP1	Nucleoid associated protein^c^	68097,59	1,59	0.070074
Transcription				
D9Q6J8_CORP1	DNA directed RNA polymerase subunit	29671,46	1,38	0.094910
D9Q748_CORP1	tRNA rRNA methyltransferase	2467,24	1,27	0.060356
D9Q8L3_CORP1	DNA directed RNA polymerase subunit omega	3784,13	−1,21	0.700214
D9Q6D1_CORP1	DNA directed RNA polymerase subunit beta	2611,89	−1,27	0.067182
D9Q8A5_CORP1	RNA polymerase-binding protein RbpA	10787,51	−1,75	0.103548
Translation				
D9Q584_CORP1	30S ribosomal protein S6	20750,74	4,82	0.047667
D9Q6E4_CORP1	Elongation factor G^b^	16882,71	3,25	0.082321
D9Q5I3_CORP1	Peptidyl prolyl cis trans isomerase^b^	61648,39	2,91	0.142641
D9Q835_CORP1	Phenylalanine tRNA ligase beta subunit	1269,7	2,74	0.064869
D9Q6L0_CORP1	50S ribosomal protein L13	5689,37	2,64	0.101816
D9Q6H2_CORP1	50S ribosomal protein L5^b^	3269,32	2,12	0.076250
D9Q918_CORP1	Proline tRNA ligase^b^	932,79	2,12	0.072151
D9Q6C0_CORP1	50S ribosomal protein L10^b^	27143,51	1,86	0.031374
D9Q6F6_CORP1	50S ribosomal protein L23^b^	6947,79	1,85	0.060878
D9Q6H1_CORP1	50S ribosomal protein L24	27887,33	1,75	0.078408
F9Y2W9_CORP1	Hypothetical protein	3152,39	1,75	0.591013
D9Q6H6_CORP1	30S ribosomal protein S8^c,b^	4941,19	1,56	0.088407
D9Q6F3_CORP1	30S ribosomal protein S10^b^	25117,55	1,54	0.048124
D9Q6G2_CORP1	50S ribosomal protein L29	2467,24	1,44	0.050948
D9Q401_CORP1	50S ribosomal protein L27^b^	2467,24	1,38	0.081399
D9Q7E8_CORP1	50S ribosomal protein L25	1358,05	−1,28	0.037225
D9Q6H8_CORP1	50S ribosomal protein L18	8920,94	−1,31	0.049024
D9Q7S4_CORP1	Homoserine dehydrogenase	698,17	−1,40	0.035138
D9Q6B7_CORP1	50S ribosomal protein L1	10218.08	−1,63	0.633387
D9Q4T4_CORP1	ATP dependent chaperone protein ClpB	1883,16	−1,80	0.045308
D9Q8N9_CORP1	Aspartate tRNA ligase	1004,33	−2,18	0.092415
D9Q7S2_CORP1	Arginine tRNA ligase	2679,11	−2,44	0.051908
Pathogenesis				
D9Q8M7_CORP1	Metallopeptidase family M24	3213,83	5,55	0.050024
D9Q608_CORP1	Penicillin binding protein transpeptidase^b^	1215,32	3,68	0.859830
D9Q827_CORP1	Metallo beta lactamase superfamily protein^c^	629,38	2,64	0.144158
D9Q721_CORP1	Hypothetical protein^c^	112025	2,24	0.260801
D9Q7K8_CORP1	Trypsin like serine protease	35041,27	1,96	0.648370
D9Q416_CORP1	ATP dependent Clp protease proteolytic^b^	2467,24	1,77	0.087255
D9Q639_CORP1	Secreted hydrolase^b^	22798,13	1,75	0.072385
D9Q588_CORP1	Penicillin binding protein^b^	9951,61	1,26	0.916125
Energy metabolism				
D9Q787_CORP1	Glucose-6-phosphate isomerase	1025,89	4,50	0.058841
D9Q7G0_CORP1	Enolase^b^	53290,95	2,18	0.068928
D9Q651_CORP1	Succinate dehydrogenase flavoprotein	797,48	2,02	0.159059
D9Q4P2_CORP1	Acetate kinase^b^	10828,79	1,96	0.063340
D9Q8G5_CORP1	Aconitate hydratase^b^	4250,81	1,85	0.217637
D9Q4Z7_CORP1	Phosphoenolpyruvate carboxykinase GTP^b^	8764,35	1,66	0.147167
D9Q7X0_CORP1	6 phosphofructokinase	1806,65	1,60	0.052885
D9Q648_CORP1	Dihydrolipoyl dehydrogenase	4110,08	1,57	0.047180
D9Q7T8_CORP1	ATP synthase subunit alpha	2467,24	1,49	0.070875
D9Q752_CORP1	Citrate synthase	6299,21	−1,21	0.116042
D9Q895_CORP1	6-Phosphogluconate dehydrogenase	4246,26	−1,89	0.050906
Lipid metabolism				
D9Q520_CORP1	Glycerophosphoryl diester phosphodieste^c^	2494,25	4,03	0.802154
D9Q718_CORP1	Methylmalonyl CoA carboxyltransferase 1^b^	2467,24	2,16	0.049504
Amino acid metabolism				
D9Q5X8_CORP1	Aspartokinase^b^	1944,81	2,86	0.043575
D9Q4C2_CORP1	Succinyl CoA Coenzyme A transferase	10894,63	1,63	0.061344
D9Q3L8_CORP1	Glutamine synthetase	320,71	−1,23	0.263700
D9Q8H7_CORP1	Cysteine desulfurase	1689,36	−1,70	0.067087
Stress response				
D9Q929_CORP1	Mycothione glutathione reductase	490,36	2,67	0.085017
D9Q5T5_CORP1	Glyoxalase Bleomycin resistance protein^c^	8420,32	2,21	0.226764
D9Q424_CORP1	DSBA oxidoreductase	12179,8	2,09	0.061566
D9Q566_CORP1	Universal stress protein A^b^	2498,69	1,70	0.034684
D9Q4P4_CORP1	Ferredoxin ferredoxin NADP reductase^b^	1086,71	1,69	0.083585
D9Q824_CORP1	Stress related protein^b^	2467,24	1,54	0.035291
D9Q692_CORP1	Thiol disulfide isomerase thioredoxin	3721,88	−2,25	0.438415
Metabolism of nucleotides and nucleic acids				
D9Q4Y6_CORP1	Deoxycytidine triphosphate deaminase	887,26	2,39	0.216897
D9Q6J1_CORP1	Adenylate kinase	15629,86	2,21	0.059568
D9Q8L4_CORP1	Guanylate kinase	2467,24	1,34	0.050095
D9Q6T2_CORP1	Ribokinase	890,09	−1,23	0.032324
D9Q4E9_CORP1	Adenylosuccinate lyase	1441,99	−1,54	0.035597
D9Q6P0_CORP1	D methionine binding lipoprotein metQ	11519,67	−1,93	0.817217
Carbohydrate metabolism				
D9Q8V2_CORP1	UDP glucose 4 epimerase^b^	2001,76	3,13	0.094403
D9Q6V6_CORP1	Phosphomannomutase ManB	1730,63	2,05	0.053146
D9Q659_CORP1	Formate acetyltransferase	5456,95	1,54	0.539548
D9Q423_CORP1	Ribose-5-phosphate isomerase B	2467,24	1,38	0.064467
D9Q6V1_CORP1	Mannose-1-phosphate guanylyltransferase	1612,45	−1,21	0.068085
Nitrogen metabolism				
D9Q4Q8_CORP1	Cytochrome c nitrate reductase small	1118,33	2,68	0.901856
Unknow function				
D9Q6T0_CORP1	Hypothetical protein	2277,6	3,62	0.050552
D9Q4R2_CORP1	Hypothetical protein	442,07	3,35	0.866986
D9Q6N1_CORP1	Hypothetical protein	561,84	3,02	0.062141
D9Q8Q4_CORP1	Hypothetical protein^c^	72711,5	2,96	0.974016
D9Q832_CORP1	Hypothetical protein	1774,59	2,90	0.752478
D9Q3S8_CORP1	Hypothetical protein^d^	837,6	2,78	0.231421
D9Q7M9_CORP1	Hypothetical protein	3246,28	2,60	0.147602
D9Q7I6_CORP1	Hypothetical protein	3751,96	2,42	0.707595
D9Q739_CORP1	Hypothetical protein	2845,77	2,28	0.836229
D9Q4C5_CORP1	Hypothetical protein	1339,3	1,83	0.023133
D9Q5C3_CORP1	Hypothetical protein	111234,6	1,49	0.946918
D9Q700_CORP1	Hypothetical protein	2467,24	1,49	0.072810
D9Q657_CORP1	Hypothetical protein	1172,66	1,41	0.830926
D9Q6F2_CORP1	Hypothetical protein	2467,24	1,34	0.061860
D9Q7X5_CORP1	Hypothetical protein	38716,45	−1,21	0.825761
D9Q4T9_CORP1	Hypothetical protein	553,76	−1,28	0.934591
D9Q6R6_CORP1	Hypothetical protein	1457,62	−1,40	0.206908
D9Q890_CORP1	Hypothetical protein	1948,52	−1,51	0.847549
D9Q6M6_CORP1	Hypothetical protein^c^	1935,68	−1,90	0.823541
Others				
D9Q6I3_CORP1	Maltotriose binding protein	5210,9	5,22	0.864851
D9Q4A3_CORP1	DsbG protein	3101,13	2,06	0.814366
D9Q6N9_CORP1	D methionine binding lipoprotein metQ	2665,58	1,79	0.764416
D9Q732_CORP1	Carbonic anhydrase^b^	689,15	1,66	0.130559
D9Q6W6_CORP1	Lipoprotein LpqB	1484,31	1,63	0.670057
D9Q556_CORP1	LSR2 like protein	2714,21	1,49	0.096802
D9Q5Q0_CORP1	UPF0145 protein	2467,24	1,37	0.025009
D9Q7W0_CORP1	Hypothetical protein	2467,24	1,26	0.039678
D9Q701_CORP1	UPF0182 protein	1682,98	1,26	0.869411
D9Q8A3_CORP1	Protein yceI^b^	16885,01	1,21	0.901679
D9Q5X4_CORP1	Serine aspartate repeat containing protein	528,36	−1,82	0.892317
D9Q826_CORP1	DoxX family protein	697,26	−2,08	0.614317
D9Q7W3_CORP1	Mycothiol acetyltransferase	947,33	−2,11	0.214833
D9Q407_CORP1	Ornithine cyclodeaminase	2566,18	−2,58	0.048247

^a^Fold change - Ratio values to: 1002Rc:11002Ct_Log(2)Ratio ≥ 1.2 proteins with *p* < 0.05

^b^Identified in an isolated of *C. pseudotuberculosis* from ovine lymph nodes [Rees et al. [12]

^c^Induced in 1002_*ovis* during to stress nitrosative [Pacheco et al. [57], Silva et al. [58]

^d^Predicted LPXTG cell wall-anchoring motif

**Table 3:** List of proteins identified in the exclusive proteome of recovered-condition

Accession	Description	Score	Biological process	SecretomeP
D9Q869_CORP1	Esterase^a^	251.44	Others	0.862935
D9Q575_CORP1	Cation transport protein	1961.29	Transport	0.062276
D9Q5N5_CORP1	Uncharacterized iron regulated membrane^a^	46.77	Transport	0.855681
D9Q3T9_CORP1	Pyridoxamine kinase	216.2	Cofactor metabolism	0.083313
D9Q751_CORP1	Phosphoserine aminotransferase	639.64	Amino acid metabolism	0.151778
D9Q537_CORP1	LytR family transcriptional regulator^a^	375.8	Transcription	0.766483
D9Q7F2_CORP1	Multicopper oxidase	74.63	Stress response	0.278840
D9Q525_CORP1	ABC transporter substrate binding lipoprotein	283.38	Transport	0.452814
D9Q6P2_CORP1	Manganese ABC transporter substrate binding^a^	236.6	Transport	0.774461
D9Q4C8_CORP1	Phosphate ABC transporter phosphate binding^a^	125.4	Transport	0.840195
D9Q4L0_CORP1	D alanyl D alanine carboxypeptidase OS	426.74	Others	0.232261
D9Q4T7_CORP1	Hyphotetical protein	157.52	Unknow function	0.349026
D9Q5A9_CORP1	Hyphotetical protein	218.02	Unknow function	0.907333
D9Q476_CORP1	Hyphotetical protein	510.32	Unknow function	0.066368
D9Q5B3_CORP1	Glucosamine-6-phosphate deaminase^b^	524,55	Carbohydrate metabolism	0.079507
D9Q474_CORP1	Glutamate racemase	343,98	Cell wall organization	0.040278
D9Q7N5_CORP1	O-methyltransferase	619,11	DNA process	0.032455
D9Q5N3_CORP1	Gamma type carbonic anhydratase	577,75	Others	0.035357
D9Q4X0_CORP1	Urease accessory protein UreD	333,12	Others	0.055896
D9Q5J0_CORP1	Phospholipase D^b^	40,25	Pathogenesis	0.409585
D9Q8S8_CORP1	Copper resistance protein CopC	4315,26	Stress response	0.964015
D9Q493_CORP1	Glutaredoxin like protein nrdH	725,98	Stress response	0.033036
D9Q6Y6_CORP1	ATP dependent RNA helicase rhlE	1438,25	Transcription	0.060627
D9Q4M0_CORP1	Cell wall channel	4008,59	Transport	0.025882
D9Q4V1_CORP1	CP40	558,79	Pathogenesis	0.926013
D9Q6V9_CORP1	Hyphotetical protein	1278,45	Unknow function	0.953803
D9Q6A8_CORP1	Hyphotetical protein	326,47	Unknow function	0.918886
D9Q485_CORP1	Hyphotetical protein	2795,11	Unknow function	0.890081
D9Q4N2_CORP1	Hypothetical protein^a^	708,75	Unknow function	0.857050
D9Q559_CORP1	Hypothetical protein^a^	475,62	Unknow function	0.472378
D9Q4L8_CORP1	Hyphotetical protein	5324,08	Unknow function	0.038893
D9Q4T0_CORP1	Hyphotetical protein	732,37	Unknow function	0.037132

^a^Induced in 1002_ovis during to stress nitrosative [Pacheco et al. [57], Silva et al. [58]

^b^Identified in an isolated of *C. pseudotuberculosis* from ovine lymph nodes [Rees et al. [12]

**Fig. 4:**
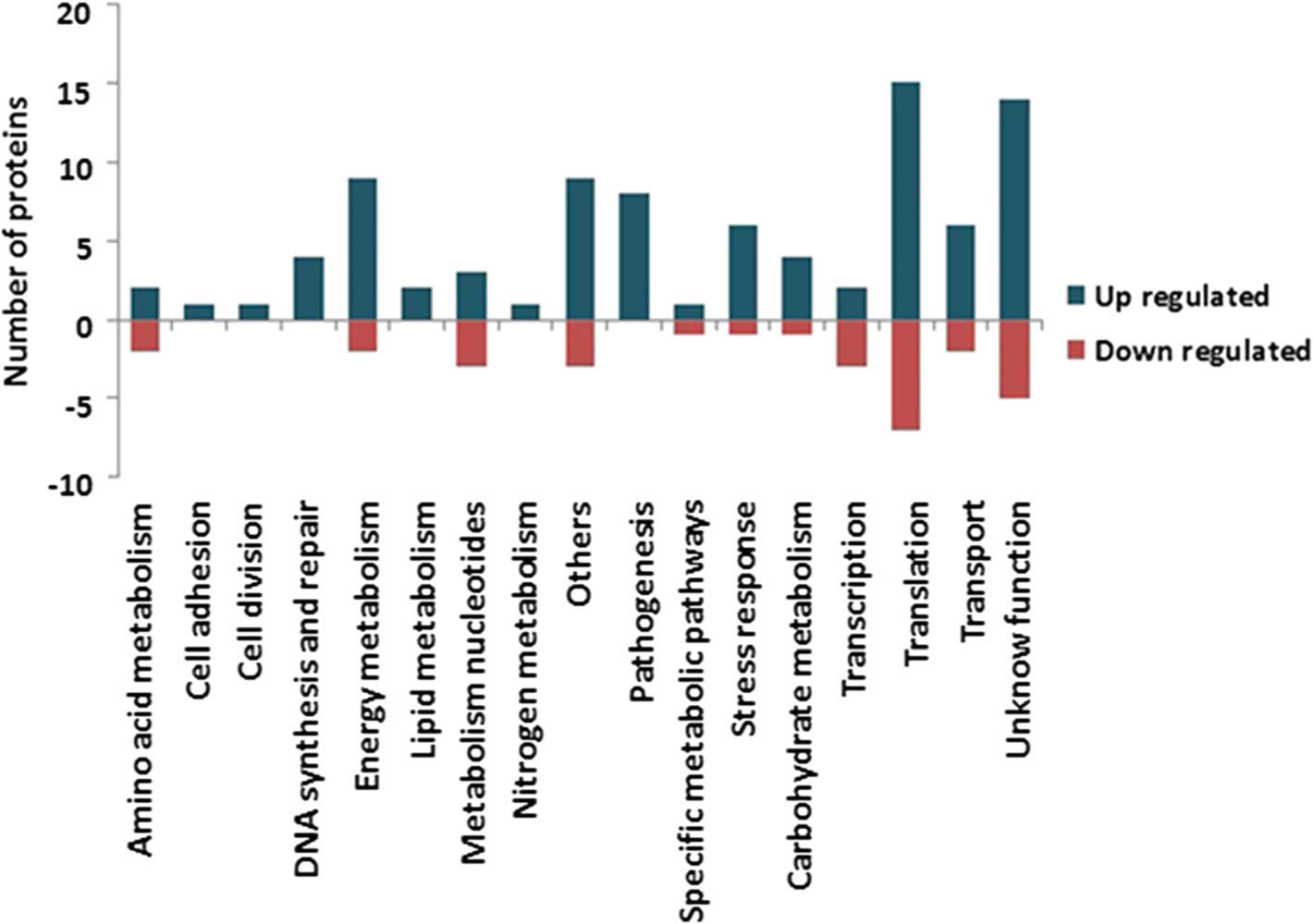
Biological processes differentially regulated in 1002_*ovis* after passage in mice. Analysis of the differentially expressed proteins grouped into biological processes for strain 1002_*ovis* after passage in mice

The proteins identified in both conditions were analyzed by SecretomeP [29] to assess whether these proteins could be exported by non-classical secretion systems. Among the expressed differentially proteins 31% (37 proteins) were predicted as secreted through non-classical secretion systems. In turn, when analyzed the exclusive proteome of each condition 19% (6 proteins) and 27% (13 proteins) were considered to be exported by non-classical secretion systems for recovered and control condition, respectively. The PIPS tool was used to evaluate whether the genes that encode the proteins which were differentially expressed and identified in the exclusive proteome of the Rc condition are included in predicted pathogenicity islands. According these analysis 16 proteins was encoded by genes located on a predicted pathogenicity island; these proteins are related to cellular metabolism, pathogenesis, transport pathway, stress response and unknown function (Additional file 4). To classify the proteins identified in functional groups, we used the Blast2Go tool [31]; according to this analysis, the proteins were grouped into 17 biological processes (Fig. 4). Among these proteins, we identified processes that are directly involved in bacterial virulence, such as protein transport, pathogenesis, cell adhesion and stress response (Table 2).

Important factors directly linked to *C. pseudotuberculosis* virulence, like the PLD phospholipase, as well as, the CP40 protease were detected only in the proteome of recovered 1002_*ovis* (Tables 1 and 3). Also, components of several secretion systems were also activated in the bacteria recovered. These include proteins related to hemin uptake, ATP-binding cassette (ABC) transporters and the Opp transporter, like OppA, OppC, and OppD. Proteins related to detoxification process were also specifically identified in the Rc supernatant: e.g. the glutaredoxin-like protein NrdH, which belongs to the NrdH-redoxins, a family of small protein disulfide oxidoreductases [34], mycothiol glutathione reductase present in Actinobacteria [35] and copper resistance protein CopC (Tables 2 and 3). In addition, we have identified 31 proteins in the recovered condition that also were detected in a strain of *C. pseudotuberculosis* isolated directly from ovine lymph nodes [12] (Tables 2 and 3). Proteins involved in the resistance to antimicrobial agents, such as penicillin-binding proteins, metallo-beta-lactamase, and penicillin-binding protein transpeptidase and proteases like Clp protease involved in the expression of cytotoxins in *Staphylococcus aureus* and *Listeria monocytogenes* [36, 37] were found induced in Rc supernatant.

## Discussion

To investigate the protein factors that could influence the adaptive processes of *C. pseudotuberculosis* biovar *ovis* during the infection process, we combined a unique bacterial passage experiment in mice with proteomic analyses of 1002*_ovis* culture supernatants, collected before and after passage. In the first analysis, we observed that strain 1002*_ovis* (isolated from caprine) exhibited a low virulence potential, which is consistent with previous reports indicating the low virulence potential of this strain [38, 39]. Although a recent in silico analysis of the 1002_*ovis* genome predicted various genes involved in virulence [40], studies examining the exoproteome of this strain under laboratory growth conditions failed to detect many of these virulence proteins (e.g., PLD exotoxin or proteins involved in the pathway of cell invasion, detoxification) [8–10].

One explanation for this relies on the fact that after being first isolated, strains 1002_*ovis* have been maintained, in vitro, under laboratory conditions with extensive passages on the culture medium, which may alter the gene expression profile of the strain, especially for effectors related to bacterial virulence. This phenomenon has also been reported in other pathogens such as *Mycobacterium bovis*, *Helicobacter pylori*, *S. aureus*, and *L. monocytogenes*. In vitro passages of these bacteria on culture medium altered both bacterial physiology and virulence profile [41–44]. However, we showed that the bacterial passage process in a murine model changed the virulence potential of strain 1002*_ovis*. Previous reports on experimental serial passages showed that pathogens such as *H. pylori*, *Escherichia coli, Xenorhabdus nematophila*, *Arcobacter butzleri*, and *Salmonella enterica* also exhibited altered virulence profiles after in vivo passage in a host, which helped identifying factors that contribute to infectious process [14–19]. Thus, as observed in these pathogens, the recovered condition also showed increased capacity to persist into host, when compared with control condition. The altered physiology and virulence status observed in 1002*_ovis* is supported by our proteomic analyses, where several proteins involved in processes favoring infection and host adaptation were differentially expressed after passage in mice.

Although our study focused on the *C. pseudotuberculosis* extracellular proteins, cytoplasmic proteins were also detected in the proteomic analyses. The presence of cytoplasmic proteins in the extracellular fraction is reported in several other proteomic studies [8–10, 12, 45]. It may be partially due to cell lysis and thus, be considered artifacts. However, cytoplasmic proteins in the culture supernatant may act as *moonlighting* proteins and be exported via a non-classical secretion pathway [30, 46]. The *moonlighting* proteins are described both Gram-positive and Gram-negative bacteria, and can be detected in different subcellular locations (cytoplasm, membrane, cell surface, and extracellular environment) and exhibit distinct functional behavior depending on the host cell type [46, 47]. Interestingly, some proteins, such as Chromosome partitioning protein ParB, Phosphoenolpyruvate carboxykinase GTP, Methylmalonyl CoA carboxyltransferase 12S subunit, Acetate kinase, and Enolase, induced in the Rc supernatants were identified only in the membrane shaving of *C. pseudotuberculosis* harvested directly from ovine lymph nodes [12].

The passage process in mice was also able to induce other proteins identified in Rc supernatants, and which contribute to the adhesion process. Proteins with an LPTXG domain, which characterizes the cell-wall anchored proteins, were identified and included monomers of membrane pilus. This latter class of proteins is described in pathogenic *Corynebacterium* species and may contribute especially in the process of cellular adhesion [48]. In *Campylobacter jejuni,* serial passages in mice induce the expression of invasiveness and increase the capacity of cell invasion [13]. Components of the Opp system were induced by the passage process, too. The Opp system facilitates the uptake of extracellular peptides, which are further used as carbon and nitrogen sources for bacterial nutrition [49]. Proteins that comprise the Opp system also were induced in a field isolated of *C. pseudotuberculosis* biovar *ovis*, when compared with the strain C231_*ovis* a laboratory reference strain [12, 50]. In the pathogen *Mycobacterium avium* the *OppA* gene was highly expressed during the infection in a mouse model [51]. We have identified known secreted virulence factors as CP40 serine protease, which previously shown to be necessary for *C. pseudotuberculosis* virulence potential and to induce an immune response [52, 53].

An important factor that precedes the chronic stage of infection by *C. pseudotuberculosis* is the capacity of this pathogen to disseminate within the host, which consequently favors the establishment of the disease [3]. In *C. pseudotuberculosis*, this process is mediated by the action of PLD exotoxin, a major virulence factor of this pathogen [54, 55] that catalyzes the dissociation of sphingomyelin and increases vascular permeability, which contributes to the dissemination process of *C. pseudotuberculosis* in the host. Here, PLD was only detected in the proteome of the Rc condition. This result is noteworthy because, a previous proteomic study performed by our research group, PLD was not identified in the extracellular proteome of 1002*_ovis* [8–10]. McKean *et al*. [5] showed that *pld* expression is expressed by different environmental factors, thus during the infection and recuperation process 1002_*ovis* was exposed to different environmental and stimulus, which may have affected the *pld* expression. A study showed that a *pld* mutant strain is indeed unable to disseminate and yields reduced virulence [55]. Here, we observed the presence of caseous lesions in different organs only at the end of experimental infection, only in the group of mice infected with the Rc condition. Altogether, the observations suggest that the expression of PLD can be modified by the passage in the host and can thus change the virulence potential of 1002_*ovis*.

Another attribute of PLD is its capacity to alter the viability of macrophage cells during the infection [5]. However, before promoting macrophages lysis, *C. pseudotuberculosis* has to be able to resist the hostile environment inside macrophages mainly against reactive oxygen species (ROS) and reactive nitrogen species (RNS). Thus, the induction of proteins involved in detoxification processes in Rc could be contributed for its resistance against ROS and RNS. The inductions of proteins related to oxidative stress also were observed in *Shigella flexneri*, after recuperation process in an in vivo infection model. We detected the mycothione glutathione reductase, a component of the mycothiol system, which is present in *Mycobacterium and Rhodococcus* genera. This system is used as an alternative mechanism of disulphide reduction and contributes to the cytosolic redox homeostasis and the resistance to ROS [35]. Glutaredoxin-like protein, NrdH, which plays an important role in the resistance to ROS, and is present in *C. glutamicum* [34] and *M. tuberculosis* [56] was also detected.

On the other hand, some proteins like dihydroxybiphenyl dioxygenase, Metallo beta lactamase superfamily protein, Formamidopyrimidine DNA glycosylase, MerR family transcriptional regulator, which were induced by 1002_*ovis* during the exposition to nitric oxide [57, 58] were also found induced in this study in the recovered condition. These proteins are related to different processes of resistance to nitrosative stress, DNA repair, antibiotic resistance, and transcription, these results show a set of proteins involved in the adaptation process of 1002_*ovis* to nitric oxide, which could contribute to the pathogenic process of this pathogen. Another type of defense of the host immune system against bacterial infection is the utilization of copper [59]. Here, CopC, a protein related to copper resistance, was detected in recovered 1002_*ovis*. In *M. tuberculosis,* proteins involved in copper resistance are essential to virulence [60, 61]. Thus, the association of this factor related to an antioxidant system with PLD could promote an effective pathway of defense against the action of the innate immune system and consequently contributes to virulence process of *C. pseudotuberculosis*.

## Conclusion

In conclusion, the virulence potential and proteomic profiles of strain 1002*_ovis* undergo dramatic changes after recovery from experimentally infected mice. The proteomic screening outlined, after the serial passage in murine model showed a set of proteins that were induced in the recovered condition. Into this group were detected known secreted virulence factors, as well as some proteins which could contribute in its virulence. Therefore, more study is necessary to show the true role of these proteins in the virulence of *C. pseudotuberculosis*. Altogether, our results demonstrate that in vitro passages alter the expression of *C. pseudotuberculosis* exoproteome leading to a reduced virulence and that a single passage in vivo, in a murine model, can induce significant changes in the *C. pseudotuberculosis* extracellular proteome, contributing to the increase in virulence of this pathogen.

## Additional files

Additional file 1: Table S1. Complete list of proteins differentially produced between the recovered and control condition of strain 1002_*ovis*. (XLSX 44 kb)

Additional file 2: Table S2. List of proteins identified in the exclusive proteome of control condition. (XLSX 12 kb)

Additional file 3: Table S3. Total list of peptide and proteins identified by LC-MS^E^. (XLSX 3 mb)

Additional file 4: Table S4. Proteins identified in the recovered condition detected in pathogenicity island. (XLSX 10 kb)
